# Mechanisms of Topoisomerase I (*TOP1*) Gene Copy Number Increase in a Stage III Colorectal Cancer Patient Cohort

**DOI:** 10.1371/journal.pone.0060613

**Published:** 2013-04-05

**Authors:** David Hersi Smith, Ib Jarle Christensen, Niels Frank Jensen, Bo Markussen, Maria Unni Rømer, Sune Boris Nygård, Sven Müller, Hans Jørgen Nielsen, Nils Brünner, Kirsten Vang Nielsen

**Affiliations:** 1 R&D, Dako A/S, Glostrup, Denmark; 2 Section of Pathobiology, Department of Veterinary Disease Biology, Faculty of Health and Medical Sciences, University of Copenhagen, Frederiksberg, Denmark; 3 Finsen Laboratory, Rigshospitalet and Biotech Research and Innovation Centre (BRIC), University of Copenhagen, Copenhagen Biocenter, Copenhagen N, Denmark; 4 Laboratory of Applied Statistics, Department of Mathematical Sciences, Faculty of Science, University of Copenhagen, Copenhagen Ø, Denmark; 5 Department of Surgical Gastroenterology 360, Hvidovre Hospital, Hvidovre, Denmark; 6 Institute of Clinical Medicine, Faculty of Health and Medical Sciences, University of Copenhagen, Copenhagen N, Denmark; The Chinese University of Hong Kong, Hong Kong

## Abstract

**Background:**

Topoisomerase I (Top1) is the target of Top1 inhibitor chemotherapy. The *TOP1* gene, located at 20q12-q13.1, is frequently detected at elevated copy numbers in colorectal cancer (CRC). The present study explores the mechanism, frequency and prognostic impact of *TOP1* gene aberrations in stage III CRC and how these can be detected by fluorescent in situ hybridization (FISH).

**Methods:**

Nine CRC cell line metaphase spreads were analyzed by FISH with a *TOP1* probe in combination with a reference probe covering either the centromeric region of chromosome 20 (CEN-20) or chromosome 2 (CEN-2). Tissue sections from 154 chemonaive stage III CRC patients, previously studied with *TOP1/*CEN-20, were analyzed with *TOP1/*CEN-2. Relationships between biomarker status and overall survival (OS), time to recurrence (TTR) in CRC and time to local recurrence (LR; rectal cancer only) were determined.

**Results:**

*TOP1* aberrations were observed in four cell line metaphases. In all cell lines CEN-2 was found to reflect chromosomal ploidy levels and therefore the *TOP1*/CEN-2 probe combination was selected to identify *TOP1* gene gains *(TOP1/*CEN-2≥1.5). One hundred and three patients (68.2%) had *TOP1* gain, of which 15 patients (14.6%) harbored an amplification (*TOP1/*CEN-20≥2.0). *TOP1* gene gain did not have any association with clinical endpoints, whereas *TOP1* amplification showed a non-significant trend towards longer TTR (multivariate HR: 0.50, p = 0.08). Once amplified cases were segregated from other cases of gene gain, non-amplified gene increases (*TOP1*/CEN-2≥1.5 and TOP1/CEN-20<2.0) showed a trend towards shorter TTR (univariate HR: 1.57, p = 0.07).

**Conclusions:**

*TOP1* gene copy number increase occurs frequently in stage III CRC in a mechanism that often includes CEN-20. Using CEN-2 as a measurement for tumor ploidy levels, we were able to discriminate between different mechanisms of gene gain, which appeared to differ in prognostic impact. *TOP1* FISH guidelines have been updated.

## Introduction

Colorectal cancer (CRC) is a leading cause of cancer death worldwide. In 2011, CRC accounted for an estimated nine percent of new cancer cases, as well as nine percent of cancer deaths in the US [1]. For the treatment of advanced CRC (stage IV), two chemotherapeutic options are available: 5-Fluorouracil (5-FU, capecitabine) in combination with irinotecan (FOLFIRI) or oxaliplatin (FOLFOX) plus biological agents. Several studies report similar response rates between the two regimens in first line treatment of advanced disease [2]–[4], with a single study reporting a significantly higher response rate with FOLFOX [5]. Interestingly, one of these studies reported a second line 6% objective response to FOLFIRI treatment following failed FOLFOX and a 21% objective response to second line FOLFOX treatment following failed FOLFIRI, indicative of non-complete cross resistance between irinotecan and oxaliplatin [4]. This finding raises the question of whether a subset of patients that received FOLFOX as first line treatment would have benefited from FOLFIRI as first line treatment, or vice versa. We therefore consider that efforts directed at the discovery of a predictive biomarker profile for FOLFOX/FOLFIRI treatment outcome are warranted.

Irinotecan, a pro-drug of SN-38, functions by inhibiting the enzyme topoisomerase I (Top1) [6]. Top1 plays an essential role in alleviating the topological stresses that arise during DNA replication and transcription by nicking, relaxing and re-ligating the double-stranded DNA structure. SN-38 binds Top1 and stabilizes the intermediate DNA-Top1 complexes. Subsequent re-ligation is inhibited, which ultimately results in cell death due to DNA damage during DNA replication or transcription [6], [7]. Top1 has due to its role as a target for SN-38 been proposed as a possible predictive biomarker for FOLFIRI treatment outcome. In advanced colorectal cancer, two large retrospective studies investigating the relationship between Top1 protein levels and irinotecan treatment outcome produce conflicting results [8], [9]. While these efforts have been directed at studying Top1 protein levels, research into chromosomal alterations involving the topoisomerase I gene (symbol: *TOP1*) are limited. Located at 20q12-q13.1, part of the frequently gained 20q region implicated in adenoma to carcinoma progression [10]–[13], *TOP1* is found at elevated copy numbers in a large fraction of stage III CRC samples when detected by Fluorescent In Situ Hybridization (FISH) [14], [15],

In our study of *TOP1*, we have previously shown that in a stage III CRC chemonaive patient cohort (n = 154), increased *TOP1* gene copy number was significantly associated with longer survival (OS) [15]. Interestingly, an estimated 71% of patients harbored a *TOP1* gene copy increase, whereas only 10% of patients harbored a *TOP1* amplification [*TOP1*/CEN-20 (centromere 20) Ratio ≥2.0] [15], indicating that gene amplification is not the most common mechanism for generating additional copies of *TOP1*. A strong correlation between *TOP1* and CEN-20 was found, revealing an association between *TOP1* and CEN-20 copy number increases. This would suggest that gene gain mechanisms involving both the *TOP1* locus and the chromosome 20 centromeric region also occur, possibly by gain of the whole 20q arm by e.g. isochromosome formation or whole chromosome 20 gain (aneusomy). This type of gene copy number increase occurs by mechanisms related to chromosome missegregation and not gene amplification. Measuring gene copy number alterations by FISH traditionally relies on the use of a same chromosome reference probe, e.g. using CEN-20 for measuring genes on chromosome 20, we therefore set out to develop a novel FISH assay to distinguish tumor specimens with *TOP1* copy number increases due to amplifications from those with increases due to 20q gain or aneusomy by applying a reference probe directed at an unrelated chromosome.

The purpose of the current study is to determine the frequency of *TOP1* alterations, map any prognostic effects of these gene aberrations, identify cut-offs that reflect the underlying genetic mechanisms of *TOP1* copy number alterations and update FISH scoring guidelines to reduce observer workload. To achieve these goals, the mechanism of *TOP1* gene copy gain was investigated in a panel of CRC cell lines with the aim of identifying a reference probe that truly reflects ploidy levels, so that *TOP1* copy number increases should be detected in relation to the total number of chromosomes (ploidy level) and this is best done through the use of a gene to centromere ratio. A novel probe combination, consisting of *TOP1* and a centromere 2-specific (CEN-2) probe, was then applied to the previously tested stage III CRC patient samples to discriminate between patients harboring *TOP1* copy number increases caused by mechanisms involving chromosome missegregation and those caused by gene amplification. The relationship between the different mechanisms of *TOP1* copy number increase and patient prognosis was investigated. Additionally, based on all FISH data, we could update the *TOP1* FISH scoring guidelines.

## Materials and Methods

### 2.1 Patients and Clinical Material

**Figure 1:**
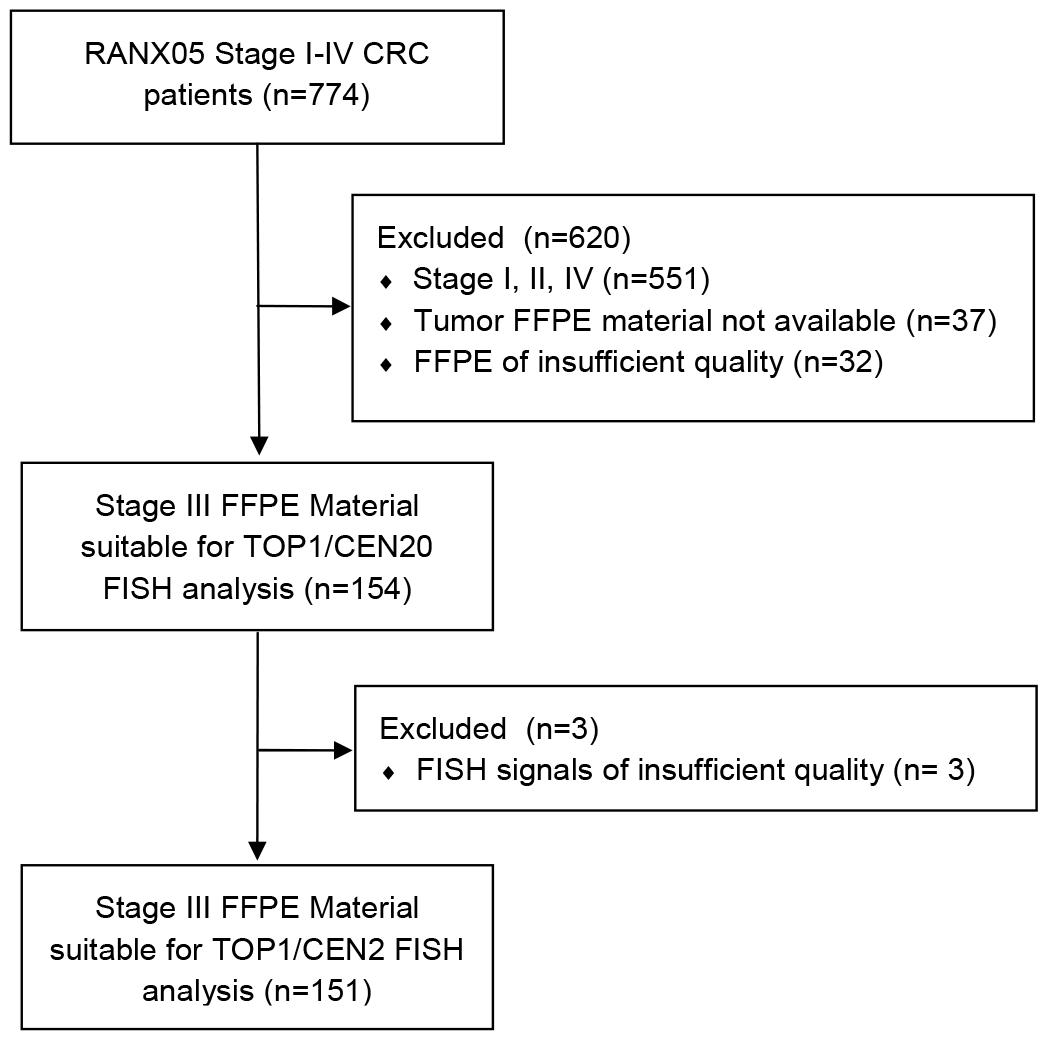
CONSORT flow diagram describing the selection method of samples included in this study.

One hundred fifty-four CRC patients with histologically verified stage III adenocarcinomas and obtainable FFPE tumor specimens were selected as previously described (see Fig. 1) [15]. All patients had surgical resections of their CRC and received no adjuvant radio- and/or chemotherapy, as this was not part of standard CRC treatment in Denmark at the time (April 1991-August 1993). Patients were randomized to receive either Ranitidine or placebo for up to five years with no effect of ranitidine on survival reported [16]. Participants provided written informed consent and the study was conducted in accordance with the Helsinki II Declaration with approval from the Danish National Board of Health (2760-419-1989), Data Protection Agency (1991-1110-751) and Central National Ethics Committee (KF 01-2045/91). The approval included collection of tissue specimens for subsequent analysis of biological markers (KF 01-078/93).

### 2.2 Preparation of Metaphases & Non-truncated Interphase Nuclei

CRC cell lines Colo-205, HCC-2998, HCT-15, HCT-116, HT-29, KM12, and SW620 were obtained from the NCI/Development Therapeutics Program, while DLD-1, LoVo, and LS-174T were obtained from the American Tissue Culture Collection. The cell lines were maintained at 37 °C, 5% CO_2_ in RPMI 1640 GlutaMAX™ growth medium (Invitrogen, Carlsbad, USA) supplemented with 10% fetal calf serum (Invitrogen). Once cultures reached a confluence of ∼70%, colcemid (Invitrogen) was added and cultures were incubated for 2 h at 37°C. Subsequently, cells were harvested and a hypotonic treatment was performed (0.075 M KCl) for 10 min. Fixation was performed (fixative: 3∶1 vol/vol absolute methanol and glacial acetic acid) and this suspension was dripped onto glass slides.

### 2.3 TOP1/CEN-2 Probe Mixture

The *TOP1* gene probe has previously been described elsewhere [14]. A CEN-2 specific probe (Dako, Glostrup, Denmark), consisting of several FITC-labeled peptide nucleic acid monomers directed at repetitive α-satellite sequences, was combined with the Texas Red-labeled DNA gene probe in the IQFISH buffer [17](Dako). The *TOP1*/CEN-20 probe combination has previously been described [14].

### 2.4 FISH Procedure

FISH reagents were from the Cytology FISH Accessory Kit (K5499) and the Histology FISH Accessory Kit (K5799) (Dako). Metaphase specimens were fixed in 3.7% formaldehyde, washed, dehydrated (in a 70%, 85%, 96% ethanol series) and air-dried. FISH probe was loaded onto slide, denatured at 82°C (*TOP1*/CEN-2∶5 min, *TOP1*/CEN-20∶10 min) and hybridized (*TOP1*/CEN-2∶1 h, *TOP1*/CEN-20: overnight). Excess probe was removed by washing in stringency buffer (*TOP1*/CEN-2∶63°C, *TOP1*/CEN-20∶65°C). Slides were washed, dehydrated, air dried and mounted. *TOP1*/CEN-2 FISH hybridization to FFPE specimens (thickness: 3 µm) was performed according to the manufacturer’s recommendations (Dako). Briefly, slides were heat pretreated (in microwave oven) and pepsin digested at 37°C. Slides were subsequently denatured at 66°C for 10 min and hybridized at 45°C for 1–2 h and thereafter treated as described above.

#### 2.4.1 Scoring

FISH signals were initially scored as previously described [14]. Briefly, signals were counted in 60 relevant non-overlapping nuclei if signals were, as a minimum, visible at 200× magnification in the appropriate filter. Scoring was performed at 1000× magnification in the Texas Red/FITC double filter. Signal counts were typed directly into an electronic scoring sheet (kindly provided by A. Schønau, Dako, Glostrup, Denmark). Initially, 60 nuclei were scored for each specimen following *TOP2A* FISH pharmDx™ guidelines (Code K5333, package insert, 1^st^ edition, 2008.01.18) where nuclei harboring both signals, as well as nuclei harboring only reference signals were scored, which facilitates the detection of gene deletions. To improve assay sensitivity, only nuclei harboring both gene- and reference signals were included for further analysis.

To determine the mechanism of *TOP1* copy number increase in cell lines, signal locations and numbers were noted for 50 metaphases for each cell line. The total number of chromosomes for each cell line was determined by taking digital images of three metaphases for each cell line and counting the total number of chromosomes manually.

To determine the haploid, diploid, triploid and tetraploid ranges for CEN-2, 60 nuclei were counted in the unaffected epithelium adjacent to tumor tissue. The diploid range was defined as follow 2n − (n/2) [min] ≤2n <2n+(n/2) [max], where 2n equals the mean CEN-2 counts per nucleus in the (diploid) unaffected epithelium. The triploid and tetraploid ranges were found by using 3n or 4n instead of 2n, respectively. The haploid and high ploidy level ranges were defined as below the diploid range and above the tetraploid ranges, respectively. Definition of ploidy ranges has previously been described [18].

### 2.5 Statistical Methods

All descriptive and survival analyses were performed by use of SAS 9.2 (SAS Institute, Cary, USA). R version 2.15.1 was used in scoring method optimization.

#### 2.5.1 Scoring methods

Gene to centromere ratios were calculated by including either the first 10 or 20 nuclei, determining *TOP1* status and comparing this to the status after inclusion of all relevant nuclei. Concordance was calculated by use of Kendall’s tau [tau = (agree-disagree)/(agree+disagree)]. Borderline intervals near the cut-off values, where additional nuclei must be included (for 10 nuclei, an additional 10 nuclei had to be scored; for 20 nuclei, an additional 20 nuclei had to be scored) were defined as greater than or equal to 1.35 (min) and less than 1.65 (max) when applied cut-off was 1.5. Using HER2/CEN-17 guidelines, the borderline interval covering the cut-off of 2.0 was defined as greater than or equal to 1.8 (min) and less than 2.2 (max) [19]. Concordance and mean number of nuclei scored were calculated with and without borderline intervals.

#### 2.5.2 Survival analysis

Three endpoints were considered: overall survival (OS, time to death by any cause), time to recurrence (TTR, time to any event related to colorectal cancer) and time to local recurrence in rectal cancer (LR) (described in detail in [15]). Kaplan-Meier estimates of survival probabilities are presented for the binary variables and some combinations. Multivariable analysis was done adjusting for gender, age (per 10 year difference in age) and tumor localization (RC versus CC). Cox regression analysis was used for the analyses. The models were validated by assessing the proportionality assumption and linearity for continuous covariates employing Schönfeld and Martingale residuals. *TOP1* and CEN-2 copy numbers, when analyzed as a continuous variable were log transformed (base 2) and therefore reflected a two-fold difference for these variates. Results are presented by hazard ratios (HR) with 95% confidence intervals (CI) and p-values. All calculated p-values were two-sided and considered significant at 0.05.

## Results

### 3.1 Mechanisms of TOP1 Gene Copy Increase in Cell Line Panel

**Figure 2:**
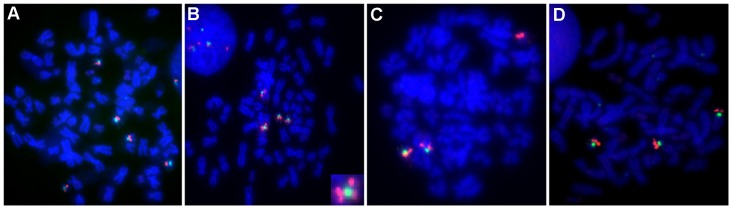
Metaphases of CRC cell lines with *TOP1* gene copy number increases. A: Colo-205, B: HT-29 (lower right corner: digitally enlarged isochromosome), C: KM12, D: SW620. Note: Due to the existence of two chromatids in each metaphase chromosome, the observed number of gene signals is double that of what is observed in an interphase nucleus.

To determine the underlying mechanism(s) of *TOP1* gene copy number increase, metaphase spreads were prepared from a panel of ten CRC cell lines. Metaphase preparation was successful for all but one of the cell lines (LS-174T). Following subsequent hybridization with the *TOP1*/CEN-20 probe, metaphase spreads were analyzed with regards to total number of chromosomes, the number of gene- and centromere-signals, as well as signal location, the results of which can be viewed in Table 1 and Figure 2. *TOP1* gene copy number increases were observed in four of the nine cell lines. In both Colo-205 and SW620, gene gain appeared to be linked to chromosome 20 aneusomy (Fig. 2A and 2D, respectively). In HT-29 (Fig. 2B), *TOP1* gene gain occurred in a fashion suggestive of 20q isochromosome formation. In KM12, *TOP1* gain occurred independently of CEN-20 (Fig. 2C). No *TOP1* amplifications were observed. As shown in Table 1, only *TOP1* gene gains which do not involve CEN-20 (in a 1∶1 fashion) are reflected in the *TOP1*/CEN-20 ratio.

**Table 1:** CRC cell line stemline populations.

Cell Line	Number of Chromosomes	Ploidy level	*TOP1* Count	CEN-20 Count	CEN-2 Counts	*TOP1*/CEN-20 Ratio	*TOP1*/CEN-2 Ratio
Colo-205	71–72	Near Triploid	5	5	3	1.00	1.67
DLD-1	45–47	Near Diploid	2	2	2	1.00	1.00
HCC-2998	46–49	Near Diploid	2	2	2	1.00	1.00
HCT-116	45–48	Near Diploid	2	2	2	1.00	1.00
HCT-15	44–46	Near Diploid	2	2	2	1.00	1.00
HT-29	68–72	Near Triploid	5	4	3	1.25	1.67
KM12	43–44	Near Diploid	3	2	2	1.50	1.50
LoVo	46–47	Near Diploid	2	2	2	1.00	1.00
SW620	43–49	Near Diploid	3	3	2	1.00	1.50

### 3.2 Identification of a New Reference Probe

To identify a relevant marker for cellular ploidy, i.e. the total number of chromosomes, the NCI and NCBI’s SKY/M-FISH and CGH Database (http://www.ncbi.nlm.nih.gov/sky/skyweb.cgi) was screened. Chromosome 2 appeared to be the least affected by independent numeric aberrations, such as whole chromosome gain, and was therefore selected for further analysis in the cell line metaphase panel. As shown in Table 1, triploid cell lines (Colo-205 and HT-29) were found to produce three CEN-2 signals, while the diploid cell lines produced only two. All *TOP1* gene copy increases were reflected in the *TOP1*/CEN-2 ratio using a cut-off value of 1.5, representing a 3∶2 situation between gene and centromere and reflecting an additional *TOP1* copy in a diploid cell.

### 3.3 Stage III CRC Patient Material

*TOP1*/CEN-2 FISH hybridization and evaluation was successful for 151 of 154 patient FFPE tumor samples (98%) (see Fig. 1). The distribution of *TOP1* and CEN-2 signals was homogeneous in tumor specimens. To improve upon sensitivity to detect specimens harboring additional copies of *TOP1*, only nuclei harboring both *TOP1* and CEN-2 signals were included in subsequent analysis, which resulted in a median of 58 nuclei scored for each tumor specimen (range: 47–60). In 50 randomly selected samples, CEN-2 signals were counted in the unaffected colon mucosa to determine mean signals counts (mean: 1.37, median: 1.38, range: 1.20–1.62). These counts were used to define the diploid range (see section 2.4.1).

In the tumor material, CEN-2 ranged from 1.19 to 2.52 with a median of 1.70. By comparing mean CEN-2 signals counts from the tumor nuclei to the non-tumor nuclei, the majority (97.4%) of tumor samples could be classified as harboring two (disomic) or three (trisomic) copies of chromosome 2 (Table 2). In the tumor samples, *TOP1* signals ranged from 1.33 to 6.72 per nucleus with a median of 3.17 signals while the *TOP1*/CEN-2 ratio ranged from 1.01 to 3.39 with a median of 1.92. No deletions (*TOP1*/CEN-2<0.8) were observed.

**Table 2:** Definition of ploidy ranges from unaffacted colon mucosa specimens and estimation of ploidy in tumor samples.

Ploidy Status	CEN-2 copy number/Whole nuclei	CEN-2 copy number/Truncated nuclei		Frequency n (%^a^)	Frequency of samples with CEN-20 aneusomy (CEN-20>CEN-2)^b^	Frequency of samples with CEN-2 aneusomy (CEN-2>CEN-20)^b^
		Mean	Range			
Haploid/Monosomy	1	0.69	<1.03	0 (0)	0 (0)	0 (0)
Diploid/Disomy	2	1.37	[1.03–1.72)	79 (52.3)	45 (29.8)	0 (0)
Triploid/Trisomy	3	2.06	[1.72–2.41)	68 (45.0)	26 (17.2)	17 (11.3)
Tetraploid/Tetrasomy	4	2.75	[2.41–3.09)	4 (2.7)	1 (0.7)	2 (1.3)
High ploidy level	>5	–	≥3.09	0 (0)	0	0 (0)

a % denotes the percentage of specimens in category relative to the total number of specimens (151).

b CEN-20 aneusomy was defined as specimens with CEN-20 signals in a higher ploidy range than CEN-2. CEN-2 aneusomy was similarly defined, although vice versa.

#### 3.3.1 Determining *TOP1* status

**Figure 3:**
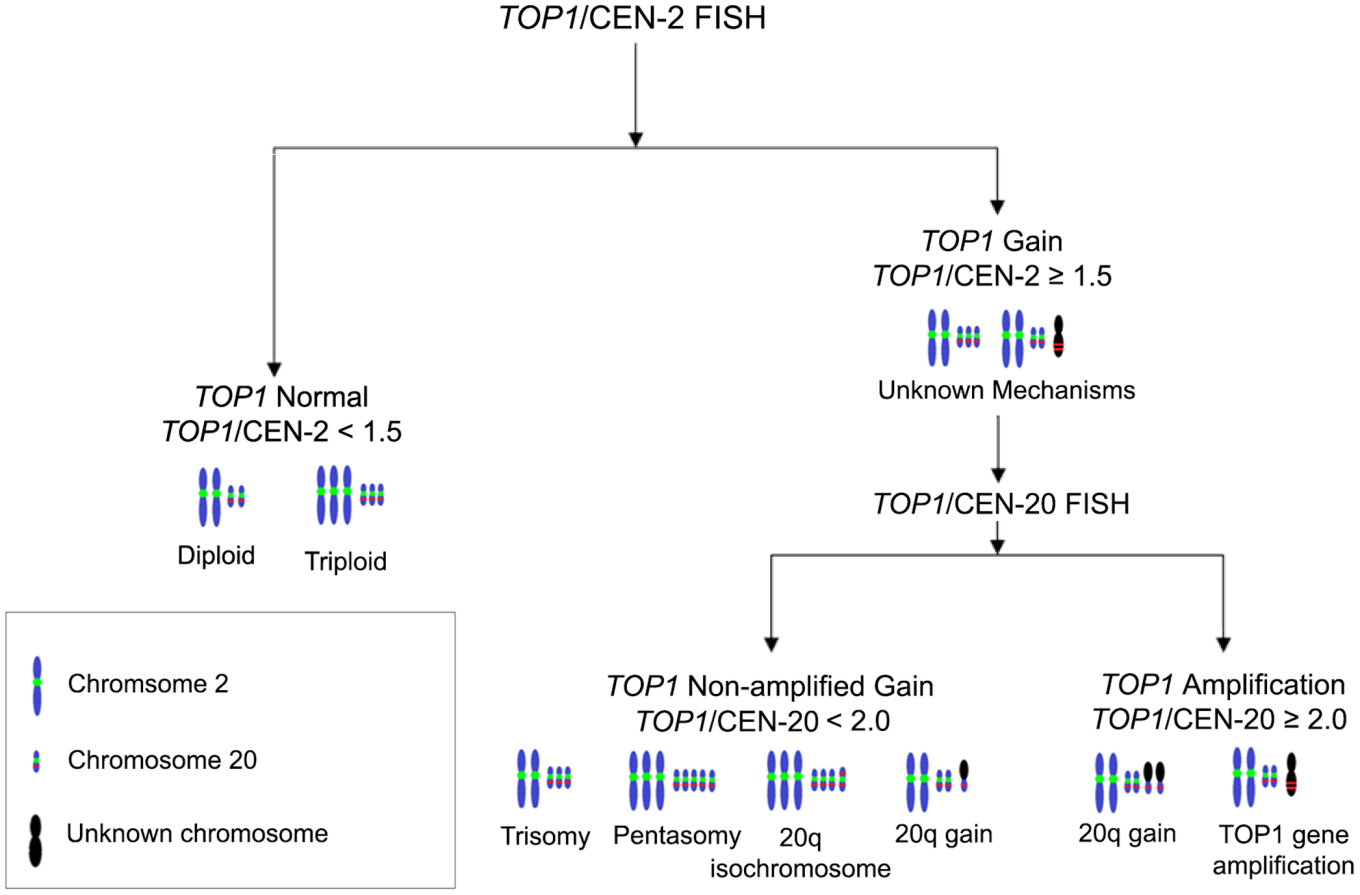
Types of *TOP1* gene copy number increases by *TOP1* status. Red line denotes *TOP1* gene signal and green dot denotes centromeric signal. Examples are based on CRC cell line metaphase results – trisomy (SW620), pentasomy (Colo-205), 20q isochromosome formation (HT-29) and 20q gain (KM12).

To identify samples harboring a *TOP1* gene copy number increase, a *TOP1*/CEN-2 ratio cut-off of 1.5 was applied. As shown in Fig. 3, samples producing ratios equal or above this cut-off received the *TOP1* status ‘Gain’, whereas those below were termed ‘*TOP1* Normal’. Initially, 103 patients (68.2%) were classified as ‘Gain’ using this cut-off.

Once tumor specimens harboring an additional copy of *TOP1* were identified, data on *TOP1*/CEN-20 was used to elucidate the mechanism of *TOP1* gene copy increase. By applying a *TOP1*/CEN-20 ratio cut-off of 2.0 to distinguish between samples where *TOP1* gene gain occurs independently of CEN-20 (*TOP1*/CEN-20≥2.0) from those where gene gain occurs due to aneusomies or 20q isochromosome formation (*TOP1*/CEN-20<2.0), specimens with a *TOP1* gain could be further dichotomized into amplified and non-amplified subgroups. Therefore, samples producing a *TOP1*/CEN-2 ratio of equal or above 1.5 and a *TOP1*/CEN-20 ratio above or equal to 2.0 were termed ‘*TOP1* Amplification’, whereas for those below 2.0 were given a ‘*TOP1* Non-amplified Gain’ status (see Fig. 3). As shown in Table 3, the majority (58.4%) of tumor specimens received a *TOP1* Non-amplified Gain status. All samples classified as *TOP1* Amplification were also found produce a *TOP1*/CEN-2 ratio of above 2.0 (see Table 3).

**Table 3:** *TOP1* status in 151 CRC samples.

*TOP1* Status	Cut-Off Values		Ratio Range (min-max)		Frequency n (%^a^)
	*TOP1*/CEN-2	*TOP1*/CEN-20	*TOP1*/CEN-2	*TOP1*/CEN-20	
Normal	<1.5	–	1.01–1.47	0.99–1.57	48 (31.8)
Non-amplified Gain	≥1.5	<2.0	1.52–3.25	0.99–1.87	88 (58.3)
Amplification	≥1.5	≥2.0	2.04–3.39	2.05–2.93	15 (10.0)

a % denotes percentage of samples relative to total number of samples.

To verify the categorization of samples into subgroups, mean CEN-2 and CEN-20 signals in tumor nuclei were compared to their respective means in unaffected colon mucosa, the results of which are listed in Table 2. Samples with mean CEN-2 and CEN-20 in the diploid range belonged in 21/34 (61.8%) cases to the *TOP1* Normal category. Near-triploid and CEN-20 aneusomic cases were most often found in the samples classified as *TOP1* Non-amplified Gain. CEN-2 aneusomy was observed in 19 (12.6%) cases.

#### 3.3.2 Association with patient outcome

The relationship between biomarker status and patient outcome was explored in both univariate and multivariate models. In this patient cohort, there were 112 deaths of all causes and 88 recurrences including cancer-specific deaths within five years [15]. *TOP1* copy number, by itself, was not significantly associated with OS, TTR or LR (see Table 4). Higher CEN-2 copy numbers were associated with better prognosis with OS as clinical endpoint in the multivariate analysis (HR: 0.38, 95% CI: 0.15–0.98, p = 0.04), while only a tendency was observed in the univariate analysis (HR: 0.49, see Table 4).

**Table 4:** Biomarker status and association to patient outcome (measured by three clinical endpoints).

Clinical Endpoint	Variate	Univariate				Multivariate*			
		HR	95% CI		p-value	HR	95% CI		p-value
OS	*TOP1*	0.77	0.53	1.13	0.18	0.76	0.51	1.13	0.18
	CEN-2	0.49	0.20	1.18	0.11	0.38	0.15	0.94	0.04
	*TOP1*/CEN-2≥1.5	0.93	0.63	1.39	0.73	0.98	0.65	1.47	0.92
	*TOP1*/CEN-2≥2.0	0.85	0.59	1.23	0.39	0.92	0.63	1.34	0.67
	*TOP1*/CEN-20≥1.5	0.92	0.61	1.37	0.67	0.78	0.51	1.18	0.23
	*TOP1*/CEN-20≥2.0	0.60	0.31	1.15	0.13	0.59	0.31	1.14	0.11
TTR	*TOP1*	0.93	0.61	1.42	0.74	0.84	0.54	1.30	0.43
	CEN-2	0.55	0.21	1.49	0.24	0.50	0.18	1.39	0.18
	*TOP1*/CEN-2≥1.5	1.40	0.87	2.25	0.17	1.31	0.80	2.14	0.28
	*TOP1*/CEN-2≥2.0	1.09	0.72	1.65	0.69	1.07	0.70	1.63	0.75
	*TOP1*/CEN-20≥1.5	0.87	0.55	1.39	0.57	0.71	0.44	1.15	0.17
	*TOP1*/CEN-20≥2.0	0.54	0.25	1.17	0.12	0.50	0.23	1.09	0.08
LR	*TOP1*	0.62	0.29	1.34	0.22	0.61	0.27	1.39	0.24
	CEN-2	0.42	0.08	2.23	0.31	0.32	0.05	2.01	0.22
	*TOP1*/CEN-2≥1.5	1.26	0.48	3.31	0.64	1.40	0.51	3.82	0.51
	*TOP1*/CEN-2≥2.0	0.74	0.36	1.54	0.43	0.77	0.37	1.63	0.50
	*TOP1*/CEN-20≥1.5	0.59	0.27	1.31	0.20	0.56	0.25	1.27	0.17
	*TOP1*/CEN-20≥2.0	NA	–	–	–	NA	–	–	–

NA: Not applicable.

* Adjusted for age, gender and tumor localization.

Initially, patients harboring *TOP1* increases (*TOP1* Gain, see Fig. 3) were compared to those without (*TOP1* Normal). Gain of *TOP1* was not significantly associated with OS or TTR in both the univariate and multivariate analysis (see Table 4). Patients with *TOP1* amplifications (*TOP1*/CEN-20≥2.0) were initially compared to non-amplified cases (*TOP1* Normal and *TOP1* Non-amplified Gain subgroups combined). Amplification of *TOP1* was not significantly associated with OS or TTR, although approached significance for TTR in the multivariate analysis (HR: 0.50, 95% CI: 0.23–1.09, p = 0.08). Analysis of *TOP1* amplifications in relation to LR failed due to a very limited number of events. Additional cut-offs for both probe combinations were investigated and the results are listed in Table 4.

**Figure 4:**
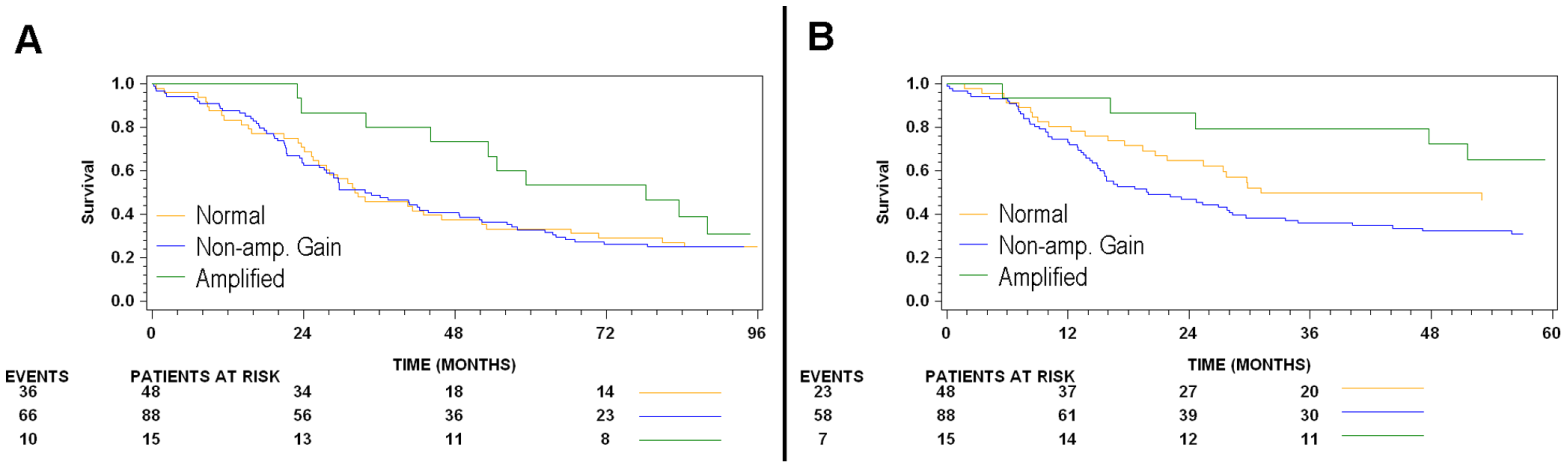
Kaplan-Meier plots illustrating patient outcome according to *TOP1* status. A (left): OS, B (right): TTR.

Following the primary analysis of data, specimens were stratified in subgroups depending on the presence and type of *TOP1* increase (see Fig. 3, listed in Table 3). As shown in Table 5, no significant difference was observed between the *TOP1* Normal, *TOP1* Non-amplified Gain and *TOP1* Amplification subgroups with OS as endpoint in both the univariate and multivariate analysis. With TTR as clinical endpoint, *TOP1* non-amplified gains showed a tendency towards shorter TTR (HR: 1.57, 95% CI: 0.97–2.55, p = 0.07) in the univariate analysis, and a similar, but weaker, tendency observed in the multivariate analysis (HR: 1.49). *TOP1* amplifications did not exhibit any significant relationship to TTR when compared to the *TOP1* Normal baseline subgroup. A Kaplan-Meier plot for these relationships can be viewed in Fig. 4B.

**Table 5:** *TOP1* Status and association to prognosis.

Clinical Endpoint	*TOP1* Status	Univariate				Multivariate*			
		HR	95% CI		p-value	HR	95% CI		p-value
OS	Normal	1.00	–	–	–	1.00	–	–	–
	Non-amplified Gain	1.01	0.68	1.52	0.95	1.08	0.71	1.64	0.72
	Amplification	0.61	0.30	1.22	0.16	0.62	0.31	1.26	0.19
TTR	Normal	1.00	–	–	–	1.00	–	–	–
	Non-amplified Gain	1.57	0.97	2.55	0.07	1.49	0.91	2.46	0.12
	Amplification	0.73	0.31	1.70	0.47	0.66	0.28	1.55	0.34

* Adjusted for age, gender and tumor localization.

### 3.4 TOP1 Scoring Guidelines

The possibility of scoring fewer nuclei in the determination of *TOP1* status presents an opportunity to diminish the observer workload. To determine whether this was possible, status of *TOP1*/CEN-2 and *TOP1*/CEN-20 was determined using 10 or 20 nuclei and compared with the ratio status after the inclusion of all relevant nuclei. As shown in Table 6, scoring a reduced number of nuclei, such as only 10 or 20 nuclei to determine *TOP1*/CEN-2 status (cut-off 1.5) classified samples with moderate concordance (0.76 and 0.91, respectively), which increased with the introduction of borderline intervals, where additional nuclei must be scored (see section 2.5.1). For the detection of *TOP1* amplifications (cut-off: 2.0), concordance was relatively high (10 nuclei: 0.96, 20 nuclei: 0.99) and was not improved with the use of borderline intervals. Additionally, alternative cut-offs were investigated, the results of which are listed in Table 6.

**Table 6:** Characteristics of the updated scoring guidelines.

Probe	Ratio cut-off	Borderline interval	Concordance		Mean number of scored nuclei	
			10 nuclei	20 nuclei	10 nuclei	20 nuclei
*TOP1*/CEN-2	1.5	none	0.76	0.91	10	20
	1.5	1.35–1.65	0.92	1.00	14.2	24.8
*TOP1*/CEN-2	2.0	none	0.70	0.80	10	20
	2.0	1.80–2.20	0.88	0.95	17.5	27.9
*TOP1*/CEN-20	1.5	none	0.82	0.87	10	20
	1.5	1.35–1.65	0.96	0.99	17.1	27.8
*TOP1*/CEN-20	2.0	none	0.96	0.99	10	20
	2.0	1.80–2.20	0.96	0.99	11	21.2

## Discussion

### 4.1 Mechanisms of TOP1 Copy Number Increase

In this study, four different mechanisms of *TOP1* copy number increase were identified. In the cell line panel mechanisms involving *TOP1* and CEN-20 (Colo-205, HT29 and SW620) as well as a mechanism where *TOP1* was gained independently of CEN-20 (KM12), were observed. In Colo-205 and SW620, chromosome 20 aneusomy was observed, in agreement with the NCI and NCBI’s SKY/M-FISH and CGH Database (http://www.ncbi.nlm.nih.gov/sky/skyweb.cgi). This type of increase transpires due to the missegregation of chromosomes during mitosis, resulting in an abnormal number of chromosomes; a karyotypic state termed ‘aneuploidy’.

In HT29, *TOP1* gain occurred in a manner suggestive of the formation of an isochromosome, in line with other results [20]. This mechanism of *TOP1* gene copy number increase occurs owing to a misdivision of the centromere (transverse breakage, rather than longitudinal) during chromosome segregation, resulting in a chromosome with two identical arms. In KM12, an additional *TOP1* signal was observed on a chromosome which did not harbor a CEN-20 signal. In the NCI and NCBI’s SKY/M-FISH and CGH Database, this cell line appears to harbor a fusion chromosome of 22q and an additional copy of 20q, where CEN-20 (or at least the alpha-satellite sequences targeted by the CEN-20 probe) was not gained along with the rest of 20q. This type of gain may be the product of chromosome 20 aneusomy followed by a Robertsonian translocation event.

While gene copy number increase can occur due to events involving larger chromosomal regions, such as the gain of whole chromosomes or chromosomal arms, it can also occur due to gene amplification. Gene amplification has been proposed to occur through several mechanisms, including errors in DNA replication and repeated breakage-fusion-bridge cycles due to double-strand DNA break or telomere dysfunction [21]–[23]. A chromosomal region preferentially gained through amplification is termed an ‘amplicon’, an approximately 0.5–10 Mb DNA fragment in length, and usually encompasses gene(s) involved in promoting tumor growth [24]. It should be noted that whole chromosome or chromosome arm alterations generally occur more frequently, but in lower magnitude, than to smaller chromosomal alterations [25].

In the present study, *TOP1* gene amplification was defined as *TOP1*/CEN-20 ratio equal or above 2.0. This definition means that *TOP1* exists at levels double that of its host chromosome. Therefore, this type of gene copy increase is probably due to the copy number increase of an amplicon and not arm-length chromosomal regions, since its mechanism of copy number increase is independent of CEN-20. No *TOP1* amplifications were observed in the nine cell lines investigated, but was detected in 10% of tumor samples. This could suggest that either this mechanism is patient-specific or that this mechanism was not present in our limited number of cell lines investigated. Amplification of the *TOP1* gene has also been reported in melanoma [26] and gastric cancer [27].

While each of the aforementioned mechanisms of *TOP1* gene copy number increase occur as single events in cell lines, our results indicate that they can occur simultaneously in tumor specimens. In 4/15 (26.7%) cases of amplification (data not shown), CEN-20 aneusomy was detected, indicative of an amplification mechanism, as well as one involving aneusomy or isochromosome formation. In samples classified as *TOP1* Non-amplified Gain, it is possible that both 20q isochromosomes and additional copies of chromosome 20 are present. However, it is only possible to classify specimens according to predominant mechanism.

### 4.2 Role of 20q

Gain of chromosome 20 or 20q has been widely reported as a recurrent chromosomal abnormality in colorectal cancer [11], [28]–[33], supporting the high frequency of *TOP1* non-amplified gains observed in this study. In vitro models suggest that 20q gain plays a causative role in tumorigenesis, as well as in increasing cellular proliferation rates [34]. In colorectal cancer, 20q is believed to play a role in colorectal adenoma to carcinoma progression [10]–[13] and is often observed in tumors exhibiting the microsatellite stable and/or chromosomal instability phenotypes [32], [35], [36]. Numerous genes located on 20q have been reported to have altered expression as a consequence 20q gain, including several well-known cancer-related genes, such as *AURKA* (20q13.2) [11], *BCL2L1* (20q11.21) [25] and *AIB1* (20q12) [27]. *TOP1* mRNA expression has not been reported to be affected by copy number increases, whereas *PLCG1*, a neighboring gene (and partly covered by the *TOP1* FISH probe), copy number increases have been found to correlate significantly with expression [27]. It should be noted that *PLCG1* appears to be involved in tumorigenesis [37].

### 4.3 Centromere 2 Versus Centromere 20

Following the analysis of *TOP1*/CEN-20 in this patient cohort [15], it became evident that *TOP1* underwent copy number increases in conjunction with CEN-20 in a large fraction of specimens. Therefore, CEN-20 was deemed to be an inappropriate marker for cellular ploidy levels. In our analysis of the metaphase cell line panel, CEN-2 was found to correctly reflect the total number of chromosomes and thus the ploidy levels. Chromosome 2 has not been reported to undergo above background numeric aberrations in CRC [11], [28]–[33], [35]. In a landmark study, consisting of 3138 cancer specimens from 26 different cancer types, 2p and 2q were shown to undergo significantly fewer arm-level alterations when compared to other chromosomes [25]. Using the Mitelman Database of Chromosome Aberrations and Gene Fusions in Cancer (http://cgap.nci.nih.gov/Chromosomes/Mitelman) to investigate the frequency of numerical aberrations involving chromosome 2 in tumors of the large intestine, 55 cases were identified from a total of 524 (consisting of adenomas, adenocarcinomas and carcinomas of the large intestine), a frequency of 10.3%. Of these 55 cases, 29 would not result in incorrect *TOP1* status; 20 would result in false negatives; 5 would result in false positives; and a single case not providing sufficient karyotypic information to determine *TOP1* status. Overall, CEN-2 would allow correct *TOP1* status categorization in 95.2% of samples. These findings support the use of CEN-2 as a reference probe to measure cellular ploidy levels in tumors of the large intestine.

A comparison of CEN-2 and CEN-20 signals in tumor nuclei revealed a high frequency of CEN-20 aneusomy (47.6%), supporting the presence of additional copies of chromosome 20 or 20q. CEN-2 aneusomy was detected in 19 specimens (12.6%). It should be noted that these findings are merely estimations, as ploidy ranges were defined by the mean signal counts in unaffected colon epithelium by two different observers. In normal tissue, mean CEN-20 was found to be 1.57 [15], whereas CEN-2 was 1.37, a discrepancy that may be attributed to interobserver variability and illustrates the potential pitfall of using mean signal counts in the analysis of FISH data.

### 4.4 Association to Patient Outcome

We have previously reported that higher *TOP1* gene copy numbers, when analyzed as a continuous variable, were associated with improved patient outcome in this particular patient cohort [15]. With OS as endpoint, *TOP1* increases were previously reported to be significantly associated with longer survival in both the univariate (HR: 0.71, 95% CI: 0.50–0.99, p = 0.04) and multivariate analysis (HR: 0.62, 95% CI: 0.42–0.90, p = 0.01) [15]; however, these findings could not be reproduced in the current study. This suggests that the previously reported findings regarding OS and TTR may have been observer dependent.

The *TOP1* Gain status, by itself, was not associated with altered OS, TTR or LR, which may be due to the inclusion of *TOP1* amplified specimens. Once amplified cases were segregated from cases of *TOP1* gain, a trend toward shorter TTR was observed for *TOP1* non-amplified gains (see Table 5). Amplification of the *TOP1* gene, representing a situation where CEN-20 is not involved in gene gain, exhibited a weak trend towards longer TTR when compared to the rest of the population, a finding which may be attributed to the low number of event in this particular subgroup. When compared only to the *TOP1* Normal subgroup, this effect diminished. While *TOP1* Non-amplified gains showed a tendency towards poor prognosis (with TTR as endpoint), *TOP1* amplification displayed a weak trend towards better prognosis (with OS as endpoint), producing non-significant hazard ratios below 1.0 (see Fig. 4B, table 5). It is unknown what effect the different *TOP1* gain mechanisms have on protein expression and why they may harbor opposite prognostic impacts. It should be noted that only 15 specimens (10%) were amplified, a low amount for a study of modest size. This observation requires validation in a larger patient cohort.

In the present study, higher CEN-2 counts were significantly associated with longer survival in the multivariate analysis (HR: 0.38, 95% CI: 0.15–0.94, p = 0.04). This suggests longer survival time for patients with tumors with a higher total number of chromosomes, i.e. near-triploidy and -tetraploidy, which stands in contrast to previously published results, which describe near-triploid karyotypes being associated with shorter survival when compared with near-diploid ones in CRC [38]. It should be noted that CEN-20 aneusomy was detected to a lesser extent at higher ploidy levels (see Table 2), opening the possibility that the association between CEN-2 and survival may be attributed to this. Alternatively, aneusomy of chromosome 2 (estimated to occur in 12.6% of specimens, detected in near-triploid and near-tetraploid tumors) may contribute to an undocumented prognostic value of chromosome 2 numeric aberrations. In any case, this result requires further validation in another patient cohort.

It is unknown which types of *TOP1* gene copy increase mechanisms, if any, yield tumors responsive or resistant to Top1 inhibitor treatment, such as irinotecan. The present study has several limitations, including a patient population treated with out-dated surgical techniques and no adjuvant therapy and should merely serve as an explorative study of the types of *TOP1* gene copy increases present in CRC and how these can be detected by FISH. Predictive biomarkers often harbor a prognostic component, a feature which may potentially overshadow the beneficial effects of a given treatment unless the prognostic element has been investigated [39]. While a limited number of significant relationships were identified in this study, we believe that the findings are relevant to report as they may aid future study design to determine whether *TOP1* gene aberrations hold any predictive value in relation to TOP1 inhibitor-based chemotherapy. We observed that *TOP1* non-amplified gains showed a non-significant tendency toward poorer prognosis, which may be attributed to increased expression of other cancer-related genes on 20q (see section 4.2). The prognostic value of amplification, which appeared to be positive, could not be clearly determined due to the low number of events. CEN-2 aneusomy occurred at levels lower than CEN-20, suggesting that it is more suitable as a reference probe to detect ploidy levels, as supported by the findings from the CRC metaphase panel. As no deletions were observed (TOP1/CEN-2<0.8), we can conclude that high level CEN-2 aneusomy does not occur independently of CEN-20 gain. The significant relationship between longer survival and higher CEN-2 counts should be interpreted with caution, as only two samples were in the tetraploid range and this relationship was not observed with other clinical endpoints. We therefore propose that CEN-2 be used in combination with other reference probes to elucidate the mechanism of gene gain, which may have relevance in other studies of copy number alterations by FISH. Future plans for *TOP1* FISH include testing both probe combinations in a retrospective material with a suitable number of irinotecan treated patients and relevant controls to determine whether any of the *TOP1* status subgroups respond to irinotecan. Furthermore, a probe set including both centromere probes in different colors will be considered to reduce observer workload.

### 4.5 Updated Guidelines

In order to reduce observer workload, the possibility of determining *TOP1* status by scoring fewer nuclei was investigated. As shown in Table 6, using a borderline interval achieves improved concordance when compared to scoring without using a borderline interval. For the validation of a HER2 assay, Wolff et al. [19] suggest a minimum of 95% concordance with an alternative validated method or same validated method, a requirement which was adopted in the current study. For *TOP1*/CEN-2 (cut-off 1.5), this requirement was surpassed by the inclusion of 20 nuclei with additional nuclei scored if the ratio was within the relevant borderline interval after 20 nuclei. This resulted in an average of 24.8 nuclei scored for each sample with these updated guidelines. For *TOP1*/CEN-20 (cut-off 2.0), scoring 10 nuclei was sufficient to achieve a concordance of 0.96. For practical purposes, we have chosen to score 20 nuclei with relevant borderline intervals to determine *TOP1* status, thereby reducing the amount of scored nuclei by 58.7 and 64.7 percent for *TOP1*/CEN-2 (cut-off 1.5) and *TOP1*/CEN-20 (cut-off 2.0), respectively (see mean nuclei scored, Table 6). We therefore propose that future use of *TOP1* FISH in CRC is based upon scoring 20 nuclei containing both gene- and reference signals and using the aforementioned borderline intervals, where an additional 20 nuclei must be scored. *TOP1* status could not be determined by use of the *TOP1* gene probe by itself in a reproducible manner (data not shown).

## References

[1] Siegel R, Ward E, Brawley O, Jemal A (2011) Cancer statistics, 2011: the impact of eliminating socioeconomic and racial disparities on premature cancer deaths. CA Cancer J Clin 61: 212–236. caac.20121 [pii];10.3322/caac.20121 [doi]. 21685461 10.3322/caac.20121

[2] Colucci G, Gebbia V, Paoletti G, Giuliani F, Caruso M et al (2005) Phase III randomized trial of FOLFIRI versus FOLFOX4 in the treatment of advanced colorectal cancer: a multicenter study of the Gruppo Oncologico Dell’Italia Meridionale. J Clin Oncol 23: 4866–4875. JCO.2005.07.113 [pii];10.1200/JCO.2005.07.113 [doi] 15939922 10.1200/JCO.2005.07.113

[3] Seymour MT, Maughan TS, Ledermann JA, Topham C, James R et al (2007) Different strategies of sequential and combination chemotherapy for patients with poor prognosis advanced colorectal cancer (MRC FOCUS): a randomised controlled trial. Lancet 370: 143–152. S0140-6736(07)61087-3 [pii];10.1016/S0140-6736(07)61087-3 [doi] 17630037 10.1016/S0140-6736(07)61087-3

[4] Tournigand C, Andre T, Achille E, Lledo G, Flesh M et al (2004) FOLFIRI followed by FOLFOX6 or the reverse sequence in advanced colorectal cancer: a randomized GERCOR study. J Clin Oncol 22: 229–237. 10.1200/JCO.2004.05.113 [doi];JCO.2004.05.113 [pii] 10.1200/JCO.2004.05.113 14657227

[5] Goldberg RM, Sargent DJ, Morton RF, Fuchs CS, Ramanathan RK et al (2004) A randomized controlled trial of fluorouracil plus leucovorin, irinotecan, and oxaliplatin combinations in patients with previously untreated metastatic colorectal cancer. J Clin Oncol 22: 23–30. 10.1200/JCO.2004.09.046 [doi];JCO.2004.09.046 [pii] 14665611 10.1200/JCO.2004.09.046

[6] Hsiang YH, Liu LF (1988) Identification of mammalian DNA topoisomerase I as an intracellular target of the anticancer drug camptothecin. Cancer Res 48: 1722–1726. 2832051

[7] Pommier Y (2009) DNA topoisomerase I inhibitors: chemistry, biology, and interfacial inhibition. Chem Rev 109: 2894–2902. 10.1021/cr900097c [doi] 19476377 10.1021/cr900097c PMC2707511

[8] Braun MS, Richman SD, Quirke P, Daly C, Adlard JW et al (2008) Predictive biomarkers of chemotherapy efficacy in colorectal cancer: results from the UK MRC FOCUS trial. J Clin Oncol 26: 2690–2698. 26/16/2690 [pii];10.1200/JCO.2007.15.5580 [doi] 18509181 10.1200/JCO.2007.15.5580

[9] Koopman M, Knijn N, Richman S, Seymour M, Quirke P et al (2009) The correlation between Topoisomerase-I (Topo1) expression and outcome of treatment with capecitabine and irinotecan in advanced colorectal cancer (ACC) patients (pts) treated in the CAIRO study of the Dutch Colorectal Cancer Group (DCCG). Eur J Cancer Suppl 7: 321.

[10] Hermsen M, Postma C, Baak J, Weiss M, Rapallo A et al (2002) Colorectal adenoma to carcinoma progression follows multiple pathways of chromosomal instability. Gastroenterology 123: 1109–1119. S0016508502002160 [pii] 12360473 10.1053/gast.2002.36051

[11] Carvalho B, Postma C, Mongera S, Hopmans E, Diskin S et al (2009) Multiple putative oncogenes at the chromosome 20q amplicon contribute to colorectal adenoma to carcinoma progression. Gut 58: 79–89. gut.2007.143065 [pii];10.1136/gut.2007.143065 [doi] 18829976 10.1136/gut.2007.143065

[12] Meijer GA, Hermsen MA, Baak JP, van Diest PJ, Meuwissen SG, et al (1998) Progression from colorectal adenoma to carcinoma is associated with non-random chromosomal gains as detected by comparative genomic hybridisation. J Clin Pathol 51: 901–909. 10070331 10.1136/jcp.51.12.901 PMC501025

[13] Hirsch D, Camps J, Varma S, Kemmerling R, Stapleton M et al (2012) A new whole genome amplification method for studying clonal evolution patterns in malignant colorectal polyps. Genes Chromosomes Cancer 51: 490–500. 10.1002/gcc.21937 [doi] 22334367 10.1002/gcc.21937 PMC3535186

[14] Romer MU, Jensen NF, Nielsen SL, Muller S, Nielsen KV et al (2012) TOP1 gene copy numbers in colorectal cancer samples and cell lines and their association to in vitro drug sensitivity. Scand J Gastroenterol 47: 68–79. 10.3109/00365521.2011.638393 [doi] 22171973 10.3109/00365521.2011.638393

[15] Romer MU, Nygard SB, Christensen IJ, Nielsen SL, Nielsen KV et al (2012) Topoisomerase 1(TOP1) gene copy number in stage III colorectal cancer patients and its relation to prognosis. Mol Oncol. S1574-7891(12)00093-2 [pii];10.1016/j.molonc.2012.09.001 [doi] 10.1016/j.molonc.2012.09.001 PMC5528401 23110915

[16] Nielsen HJ, McArdle C, Moesgaard F, The RANX05 Study Group (1998) The effect of ranitidine on long-term survival on primary colorectal cancer. A 40 months interim analysis. GI Cancer 227–233.

[17] Matthiesen SH, Hansen CM (2012) Fast and Non-Toxic In Situ Hybridization without Blocking of Repetitive Sequences. PLoS One 7: e40675 10.1371/journal.pone.0040675 [doi];PONE-D-12-07283 [pii] 22911704 10.1371/journal.pone.0040675 PMC3404051

[18] Nielsen KV, Ejlertsen B, Moller S, Jensen MB, Balslev E et al (2012) Lack of independent prognostic and predictive value of centromere 17 copy number changes in breast cancer patients with known HER2 and TOP2A status. Mol Oncol 6: 88–97. S1574-7891(11)00142-6 [pii];10.1016/j.molonc.2011.11.006 [doi] 22153616 10.1016/j.molonc.2011.11.006 PMC5528381

[19] Wolff AC, Hammond ME, Schwartz JN, Hagerty KL, Allred DC et al (2007) American Society of Clinical Oncology/College of American Pathologists guideline recommendations for human epidermal growth factor receptor 2 testing in breast cancer. J Clin Oncol 25: 118–145. JCO.2006.09.2775 [pii];10.1200/JCO.2006.09.2775 [doi] 17159189 10.1200/JCO.2006.09.2775

[20] Boonsong A, Marsh S, Rooney PH, Stevenson DA, Cassidy J et al (2000) Characterization of the topoisomerase I locus in human colorectal cancer. Cancer Genet Cytogenet 121: 56–60. S0165-4608(00)00242-9 [pii] 10958942 10.1016/s0165-4608(00)00242-9

[21] Myllykangas S, Knuutila S (2006) Manifestation, mechanisms and mysteries of gene amplifications. Cancer Lett 232: 79–89. S0304-3835(05)00827-X [pii]; 10.1016/j.canlet.2005.07.045 [doi] 16288831 10.1016/j.canlet.2005.07.045

[22] Albertson DG, Collins C, McCormick F, Gray JW (2003) Chromosome aberrations in solid tumors. Nat Genet 34: 369–376. 10.1038/ng1215 [doi];ng1215 [pii] 12923544 10.1038/ng1215

[23] Albertson DG (2006) Gene amplification in cancer. Trends Genet 22: 447–455. S0168-9525(06)00175-2 [pii];10.1016/j.tig.2006.06.007 [doi] 16787682 10.1016/j.tig.2006.06.007

[24] Lengauer C, Kinzler KW, Vogelstein B (1998) Genetic instabilities in human cancers. Nature 396: 643–649. 10.1038/25292 [doi] 9872311 10.1038/25292

[25] Beroukhim R, Mermel CH, Porter D, Wei G, Raychaudhuri S et al (2010) The landscape of somatic copy-number alteration across human cancers. Nature 463: 899–905. nature08822 [pii];10.1038/nature08822 [doi] 20164920 10.1038/nature08822 PMC2826709

[26] Ryan D, Rafferty M, Hegarty S, O’Leary P, Faller W et al (2010) Topoisomerase I amplification in melanoma is associated with more advanced tumours and poor prognosis. Pigment Cell Melanoma Res 23: 542–553. PCR720 [pii];10.1111/j.1755-148X.2010.00720.x [doi] 20465595 10.1111/j.1755-148X.2010.00720.x

[27] Fan B, Dachrut S, Coral H, Yuen ST, Chu KM et al (2012) Integration of DNA copy number alterations and transcriptional expression analysis in human gastric cancer. PLoS One 7: e29824 10.1371/journal.pone.0029824 [doi];PONE-D-11-18525 [pii] 22539939 10.1371/journal.pone.0029824 PMC3335165

[28] Ried T, Knutzen R, Steinbeck R, Blegen H, Schrock E et al (1996) AID-GCC5>3.0.CO;2-2 [pii]; 10.1002/(SICI)1098-2264(199604)15:4<234::AID-GCC5>3.0.CO;2-2 [doi] 10.1002/(SICI)1098-2264(199604)15:4<234::AID-GCC5>3.0.CO;2-2 8703849

[29] Rooney PH, Boonsong A, McKay JA, Marsh S, Stevenson DA et al (2001) Colorectal cancer genomics: evidence for multiple genotypes which influence survival. Br J Cancer 85: 1492–1498. 10.1054/bjoc.2001.2095 [doi];S0007092001920956 [pii] 11720434 10.1054/bjoc.2001.2095 PMC2363933

[30] Korn WM, Yasutake T, Kuo WL, Warren RS, Collins C et al (1999) AID-GCC2>3.0.CO;2-6 [pii] 10.1002/(sici)1098-2264(199906)25:2<82::aid-gcc2>3.0.co;2-6 10337990

[31] Tsafrir D, Bacolod M, Selvanayagam Z, Tsafrir I, Shia J et al (2006) Relationship of gene expression and chromosomal abnormalities in colorectal cancer. Cancer Res 66: 2129–2137. 66/4/2129 [pii];10.1158/0008-5472.CAN-05-2569 [doi] 16489013 10.1158/0008-5472.CAN-05-2569

[32] Jones AM, Douglas EJ, Halford SE, Fiegler H, Gorman PA et al (2005) Array-CGH analysis of microsatellite-stable, near-diploid bowel cancers and comparison with other types of colorectal carcinoma. Oncogene 24: 118–129. 1208194 [pii];10.1038/sj.onc.1208194 [doi] 15531920 10.1038/sj.onc.1208194

[33] De Angelis PM, Clausen OP, Schjolberg A, Stokke T (1999) Chromosomal gains and losses in primary colorectal carcinomas detected by CGH and their associations with tumour DNA ploidy, genotypes and phenotypes. Br J Cancer 80: 526–535. 10.1038/sj.bjc.6690388 [doi] 10408863 10.1038/sj.bjc.6690388 PMC2362312

[34] Tabach Y, Kogan-Sakin I, Buganim Y, Solomon H, Goldfinger N et al (2011) Amplification of the 20q chromosomal arm occurs early in tumorigenic transformation and may initiate cancer. PLoS One 6: e14632 10.1371/journal.pone.0014632 [doi] 21297939 10.1371/journal.pone.0014632 PMC3031497

[35] Dyrso T, Li J, Wang K, Lindebjerg J, Kolvraa S et al (2011) Identification of chromosome aberrations in sporadic microsatellite stable and unstable colorectal cancers using array comparative genomic hybridization. Cancer Genet 204: 84–95. S0165-4608(10)00482-6 [pii];10.1016/j.cancergencyto.2010.08.019 [doi] 21504706 10.1016/j.cancergencyto.2010.08.019

[36] Lassmann S, Weis R, Makowiec F, Roth J, Danciu M et al (2007) Array CGH identifies distinct DNA copy number profiles of oncogenes and tumor suppressor genes in chromosomal- and microsatellite-unstable sporadic colorectal carcinomas. J Mol Med (Berl) 85: 293–304. 10.1007/s00109-006-0126-5 [doi] 17143621 10.1007/s00109-006-0126-5

[37] Markova B, Albers C, Breitenbuecher F, Melo JV, Brummendorf TH et al (2010) Novel pathway in Bcr-Abl signal transduction involves Akt-independent, PLC-gamma1-driven activation of mTOR/p70S6-kinase pathway. Oncogene 29: 739–751. onc2009374 [pii];10.1038/onc.2009.374 [doi] 19881535 10.1038/onc.2009.374

[38] Bardi G, Fenger C, Johansson B, Mitelman F, Heim S (2004) Tumor karyotype predicts clinical outcome in colorectal cancer patients. J Clin Oncol 22: 2623–2634. 10.1200/JCO.2004.11.014 [doi];22/13/2623 [pii] 15226330 10.1200/JCO.2004.11.014

[39] Nielsen KV, Brunner N (2011) Re: Topoisomerase II alpha and responsiveness of breast cancer to adjuvant chemotherapy. J Natl Cancer Inst 103: 352–353. djq528 [pii];10.1093/jnci/djq528 [doi] 21217082 10.1093/jnci/djq528

